# Pharmacodynamic evaluation of BRII-693, a next-generation synthetic macrocyclic peptide antibiotic, in the neutropenic mouse thigh and lung infection models against gram-negative pathogens

**DOI:** 10.1128/aac.01880-25

**Published:** 2026-02-26

**Authors:** Alexander J. Lepak, Yali Zhu, David R. Andes

**Affiliations:** 1 Department of Medicine, University of Wisconsin School of Medicine and Public Health 200889, Madison, Wisconsin, USA; 2 Brii Biosciences Department of Clinical Pharmacology, Brii Biosciences 733165 , Durham, North Carolina, USA; 3 Department of Medical Microbiology and Immunology, University of Wisconsin 732057, Madison, Wisconsin, USA; University of Houston, Houston, Texas, USA

**Keywords:** BRII-693, pharmacodynamics, gram-negative

## Abstract

BRII-693 is a next-generation synthetic macrocyclic peptide antibiotic for infections caused by drug-resistant gram-negative pathogens. This study evaluated the pharmacodynamic activity of BRII-693 against common gram-negative pathogens using neutropenic mouse thigh (11 strains) and lung (15 strains) infection models. BRII-693 exhibited dose-dependent increases in pharmacokinetic exposure in both plasma and epithelial lining fluid (ELF). Due to its prolonged elimination half-life within ELF, the ELF area under the curve (AUC) was higher than plasma AUC on a mg/kg basis. In dose-ranging PK/PD studies, BRII-693 demonstrated dose-dependent antibacterial activity. In both the thigh and lung models, bacterial stasis was achieved at 24 h plasma AUC/MIC values of 1–19 for *Escherichia coli*, *Klebsiella pneumoniae*, and *Acinetobacter baumannii*, and 1-log kill required only modestly higher targets given the steep exposure-response relationships. Higher target exposures were noted for *Pseudomonas aeruginosa* studies. In the lung model, BRII-693 achieved robust cidal endpoints against all tested organisms. Maximal effect (3–4 log reduction in bacterial burden) converged at a 24 h plasma AUC/MIC value of 50. These findings demonstrate robust PK/PD profile and *in vivo* efficacy of BRII-693 against clinically relevant gram-negative pathogens, supporting its potential as a next-generation macrocyclic peptide antibiotic.

## INTRODUCTION

Infections caused by gram-negative organisms, in particular *Enterobacterales* spp., *Acinetobacter* spp., and *Pseudomonas aeruginosa*, are a leading cause of morbidity and mortality in hospitalized patients (1). In part, this is due to the enhanced ability for many of these pathogens to develop multiple drug resistance mechanisms. Recent therapeutic advances, including novel beta-lactam/beta-lactamase (BL/BLI) combinations, enhanced tetracycline derivatives, and cefiderocol, have expanded treatment options for drug-resistant gram-negative infections. However, concerns remain regarding the sustainability of these therapies, as resistance to these new agents is not uncommon; clinical outcomes are often suboptimal; and many therapies are ineffective against certain strains (e.g., metallo-β-lactamases, multi-drug-resistant *P. aeruginosa* or *Acinetobacter* spp.) (2–7).

While the development of drugs with novel mechanisms of action is promising, another strategy is to innovate proven drugs with known limitations. Polymyxin B is one such candidate, offering broad-spectrum activity against gram-negative pathogens, including those resistant to other therapies. Its clinical utility, however, is limited by exposure-related toxicities yielding a narrow therapeutic window and reduced efficacy in pulmonary infections due to surfactant binding (8–11). BRII-693 (formerly QPX9003 and F365) is a synthetic lipopeptide variant of polymyxin B designed to overcome these limitations (10). Structural modifications at non-conserved positions within the polymyxin scaffold confer enhanced potency against gram-negative pathogens, notably *P. aeruginosa* and *A. baumannii*, in both *in vitro* and *in vivo* models and, unlike polymyxin B, permit full activity in the presence of pulmonary surfactant (10). Importantly, BRII-693 offers a wider therapeutic window and a decreased potential for nephrotoxicity compared to traditional polymyxin compounds (10).

Pharmacokinetic/pharmacodynamic (PK/PD) studies are an integral step in early drug development (12, 13). These studies integrate pharmacokinetic parameters, organism susceptibility, and time-dependent efficacy to define exposure-response relationships for a candidate molecule (14). The goal is to define exposure-response relationships and identify pharmacodynamic targets that correlate with efficacy in animal models. These targets inform optimized dosing strategies for Phase II/III clinical trials based on human PK data and *in vitro* susceptibility distributions. This study evaluates the exposure-response relationship for BRII-693 using the murine thigh and lung infection models, providing foundational data to support clinical development.

## RESULTS

### Organisms and *in vitro* susceptibility testing

The study organisms and susceptibility testing results are shown in Table 1. The BRII-693 MIC varied eightfold (range 1–8 mg/L).

**TABLE 1:** Study organisms and BRII-693 susceptibility results

Organism	BRII-693 MIC (mg/L)	Ceftazidime MIC (mg/L)	Meropenem MIC (mg/L)
*A. baumannii* 1064791	2	8	64
*A. baumannii* 1170594	2	8	0.5
*A. baumannii* 1082759	1	2	0.125
*A. baumannii* 1109046	2	4	0.125
*E. coli* ATCC 25922	1	0.125	0.5
*E. coli* 1-894-1	1	0.5	NA^*a*^
*E. coli* 2671	2	2	4
*K. pneumoniae* ATCC 43816	2	0.125	1
*K. pneumoniae* ATCC 4110	8	NA	0.5
*K. pneumoniae* 2693	1	NA	0.5
*K. pneumoniae* 2697	1	NA	16
*P. aeruginosa* 27853	1	0.5	0.125
*P. aeruginosa* 4304A	2	0.5	0.25
*P. aeruginosa* 2757	1	1	4
*P. aeruginosa* 24530	1	NA	8
*P. aeruginosa* 823	1	4	0.25
*P. aeruginosa* 3076	2	16	4

^*a*^ NA, not available.

### Pharmacokinetics of BRII-693 in mice

**Fig 1:**
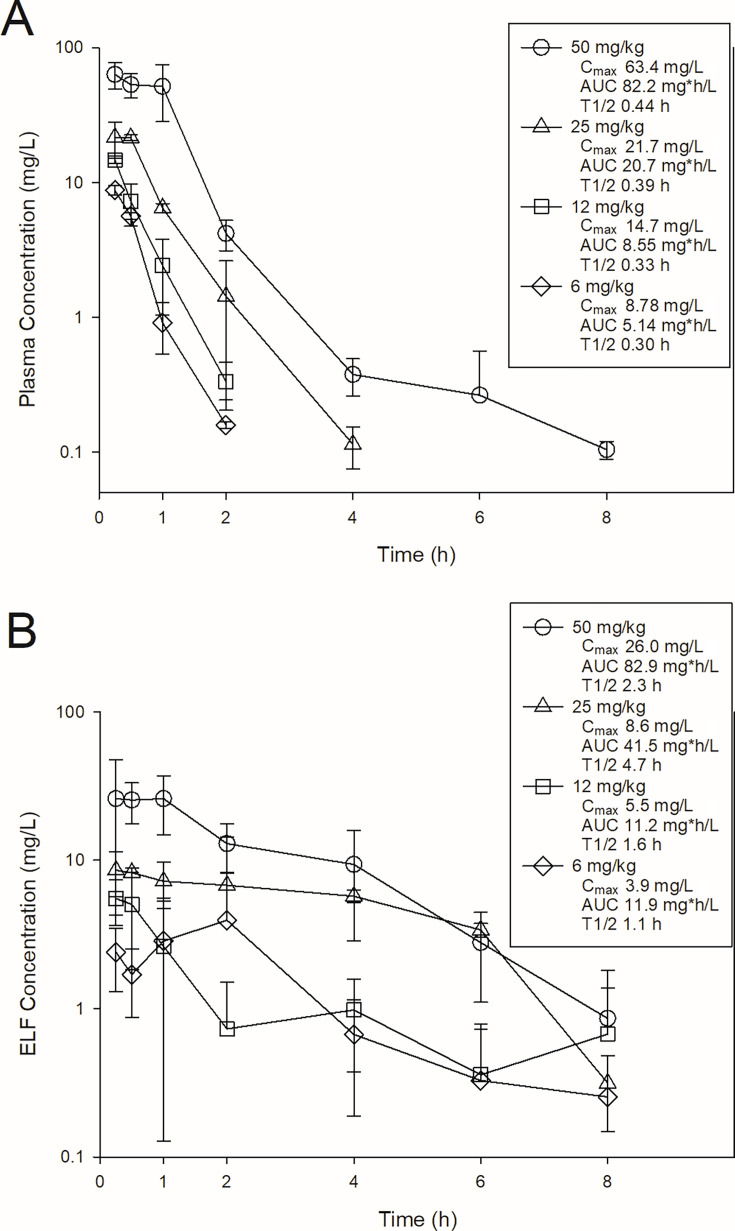
Single-dose plasma (**A**) and ELF (**B**) pharmacokinetics of BRII-693 in mice. Four different doses of BRII-693 were administered to mice by intraperitoneal route. Groups of three mice were sampled at each time point. Each symbol represents the mean from three animals; error bars show standard deviations. Shown in the symbol key are the maximum concentration (C_max_), the area under the concentration curve from 0 h to infinity (AUC), and the terminal elimination half-life (T_1/2_).

The time course of BRII-693 concentrations in plasma and epithelial lining fluid (ELF) is shown in Fig. 1. After intraperitoneal (IP) administration of BRII-693 at 6, 12, 25, and 50 mg/kg, the plasma C_max_ ranged from 8.78 to 63.4 mg/L, area under the curve (AUC)_0–∞_ from 5.14 to 82.2 mg*h/L, and elimination half-life (T_½_) from 0.3 to 0.44 h. Corresponding ELF C_max_ ranged from 3.9 to 26.0 mg/L, AUC_0–∞_ from 11.9 to 82.9 mg*h/L, and elimination T_½_ from 1.1 to 4.7 h. Compared with plasma, ELF demonstrated markedly longer elimination half-life leading to higher AUC exposures on a mg/kg basis.

### PK/PD magnitude determination for BRI-693 in the thigh model

**Fig 2:**
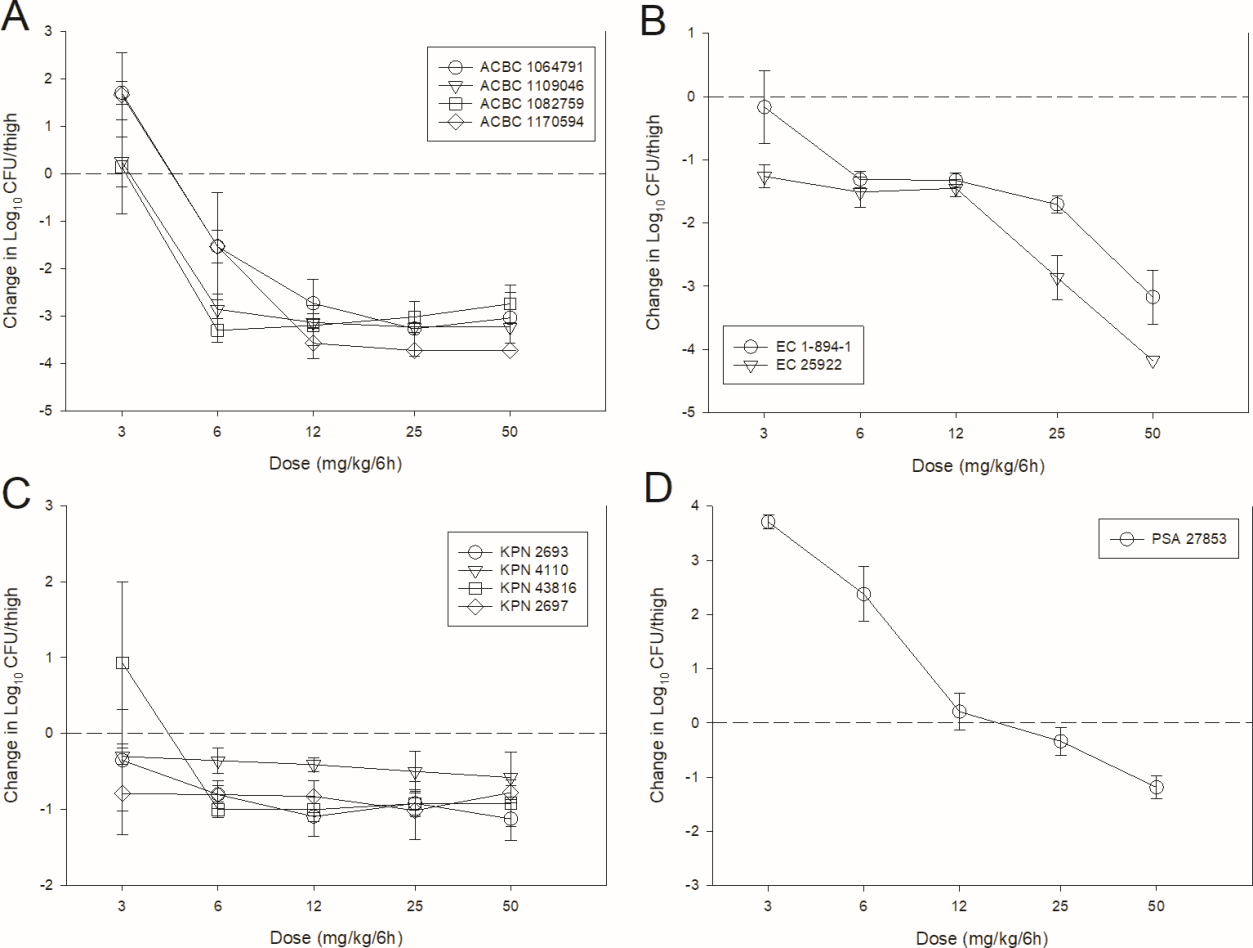
BRII-693 dose-response curves in the mouse thigh infection model against *A. baumannii* (**A**), *E. coli* (**B**), *K. pneumoniae* (**C**), and *P. aeruginosa* (**D**). Five total dose levels were administered intraperitoneally in a 6-hourly regimen to thigh-infected mice. The change in burden was assessed over a 24-h treatment duration. Points above the dashed line represent net increase in bacterial burden, whereas those below the dashed line represent a net decrease in bacterial burden.

**Fig 3:**
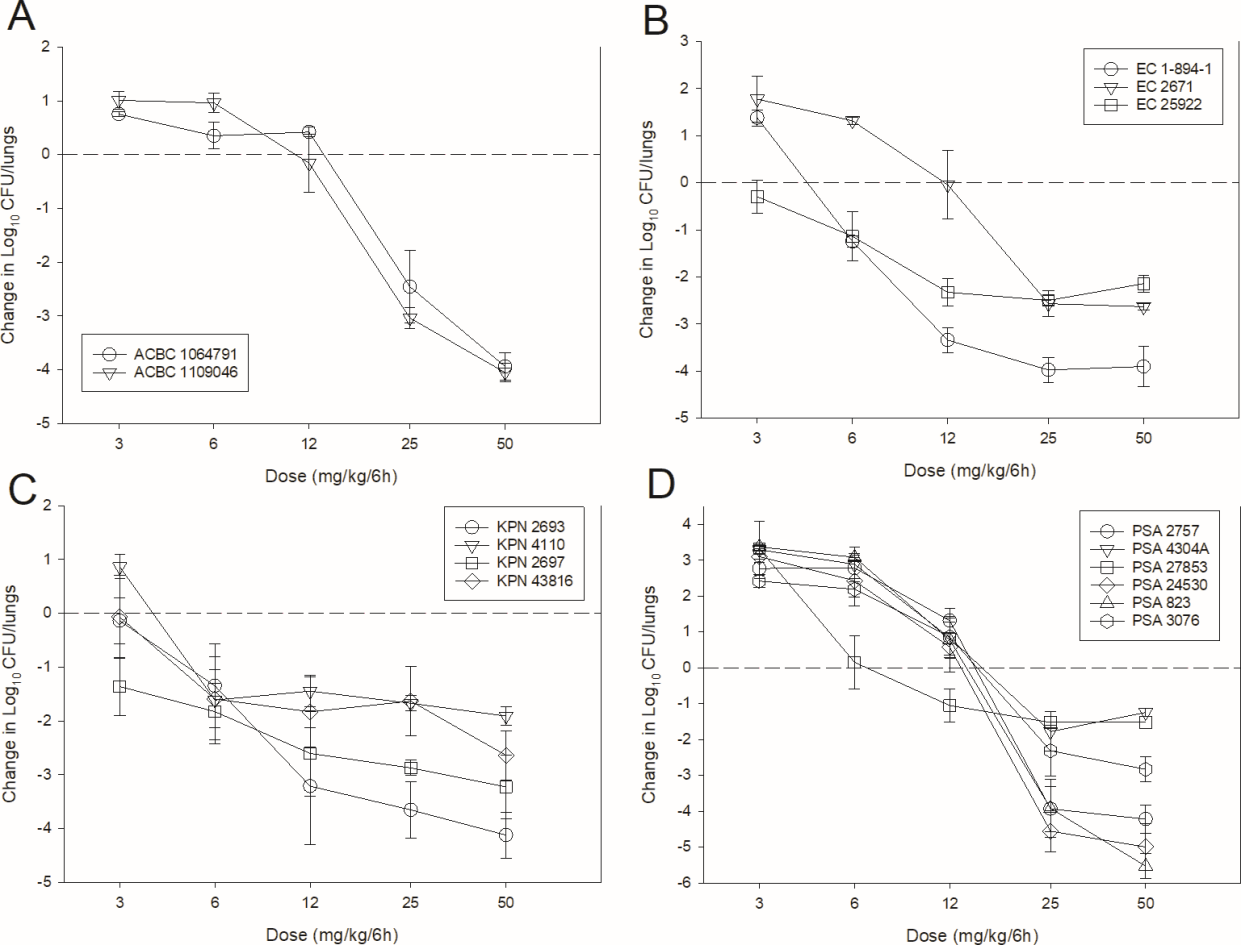
BRII-693 dose-response curves in the mouse lung infection model against *A. baumannii* (**A**), *E. coli* (**B**), *K. pneumoniae* (**C**), and *P. aeruginosa* (**D**). Five total dose levels were administered intraperitoneally in a 6-hourly regimen to lung-infected mice. The change in burden was assessed over a 24-h treatment duration. Points above the dashed line represent net increase in bacterial burden, whereas those below the dashed line represent a net decrease in bacterial burden.

The dose-response curves for BRII-693 against multiple gram-negative pathogens in the neutropenic mouse thigh (11 total experiments) and lung (15 total experiments) infection models are shown in Fig. 2 and 3, respectively. At the start of treatment, bacterial burdens were 6.0 ± 0.5 log_10_ CFU/thigh and 7.1 ± 1.1 log_10_ CFU/lungs. Growth in untreated controls was >1 log_10_ in all but one experiment and relatively similar within species groups (Table 2).

**TABLE 2:** BRII-693 pharmacodynamic targets associated with net stasis, 1-log kill, and 2-log kill in the neutropenic murine thigh and lung infection models^*a*^

				Dose (mg/kg/6h)			Plasma^*b*^			ELF		
Species/model	Strain	MIC	Log control growth	Static dose	1-Log kill dose	2-Log kill dose	Static dose 24 h AUC/MIC	1-Log kill 24 h AUC/MIC	2-Log kill 24 h AUC/MIC	Static dose 24 h AUC/MIC	1-Log kill 24 h AUC/MIC	2-Log kill 24 h AUC/MIC
*A. baumannii* lung	1064791	2	1.05	13.13	17.74	22.67	19.19	27.80	36.99	26.26	47.21	69.57
	1109046	2	1.22	10.48	14.16	17.97	15.37	21.12	28.23	21.72	30.94	48.25
*A. baumannii* thigh	1064791	2	3.19	4.34	5.33	6.96	7.43	9.13	11.37			
	1109046	2	1.94	2.66	3.12	3.63	4.56	5.34	6.21			
	1082759	1	2.87	3.05	3.45	3.99	10.45	11.83	13.66			
	1170594	2	2.83	4.45	5.36	6.59	7.62	9.17	10.94			
*E. coli* lung	1-894-1	1	1.68	4.71	5.80	7.11	16.12	19.87	23.08	36.70	45.25	45.97
	2671	2	2.05	10.13	13.99	20.25	14.97	20.81	32.47	21.85	30.20	58.59
	25922	1	2.81	2.71	4.58	11.08	9.30	15.68	32.09	21.17	35.70	43.00
*E. coli* thigh	1-894-1	1	2.17	2.26	6.57	19.68	7.76	21.86	62.81			
	25922	1	2.77	1.44	3.49	8.78	4.95	11.94	26.86			
*K. pneumoniae* lung	2693	1	1.19	2.94	4.81	7.32	10.07	16.47	23.55	22.92	37.50	45.81
	4110	8	1.87	3.68	4.61	>50	1.58	1.97	NA	3.59	4.49	NA
	2697	1	0.88	0.29	1.22	3.65	1.01	4.17	12.50	2.29	9.50	28.46
	43816	2	3.75	2.96	8.05	36.48	5.06	12.61	97.76	11.53	22.63	118.00
*K. pneumoniae* thigh	2693	1	3.72	0.89	18.42	>50	3.06	58.11	NA			
	4110	8	2.34	<0.29	>50	>50	NA	NA	NA			
	43816	2	2.94	3.92	6.41	>50	6.71	10.74	NA			
	2697	1	1.11	<0.29	24.90	>50		41.13				
*P. aeruginosa* lung	2757	1	3.38	13.88	15.43	17.29	41.21	46.97	53.90	59.37	73.39	90.28
	4304A	2	3.51	13.99	19.32	>50	20.81	30.74	NA	30.18	54.37	NA
	27853	1	4.46	6.35	9.51	>50	21.34	28.52	NA	46.54	44.17	NA
	24530	1	3.30	12.65	14.34	16.27	36.61	42.93	50.11	48.19	63.56	81.05
	823	1	3.33	13.44	15.50	17.81	39.57	47.25	55.87	55.38	74.08	95.06
	3076	2	2.31	13.35	16.37	20.79	19.62	25.25	33.47	27.28	41.00	61.00
*P. aeruginosa* thigh	27853	1	4.06	14.65	34.54	>50	44.05	176.51	NA			

^*a*^ NA, not achieved.

^*b*^ Plasma targets are shown using total drug concentrations. As protein binding is 36% in mice for BRII-693, the free drug values would be estimated to be approximately 64% of the shown plasma AUC/MIC values.

In both models, increasing doses of BRII-693 exhibited increased antibacterial effects; however, thigh experiments with *K. pneumoniae* showed relatively shallow dose-response curves in three of four experiments. At the highest doses, net log reduction endpoints were achieved against all strains, and lung infection experiments demonstrated impressive bactericidal endpoints (≥2-log kill).

**Fig 4:**
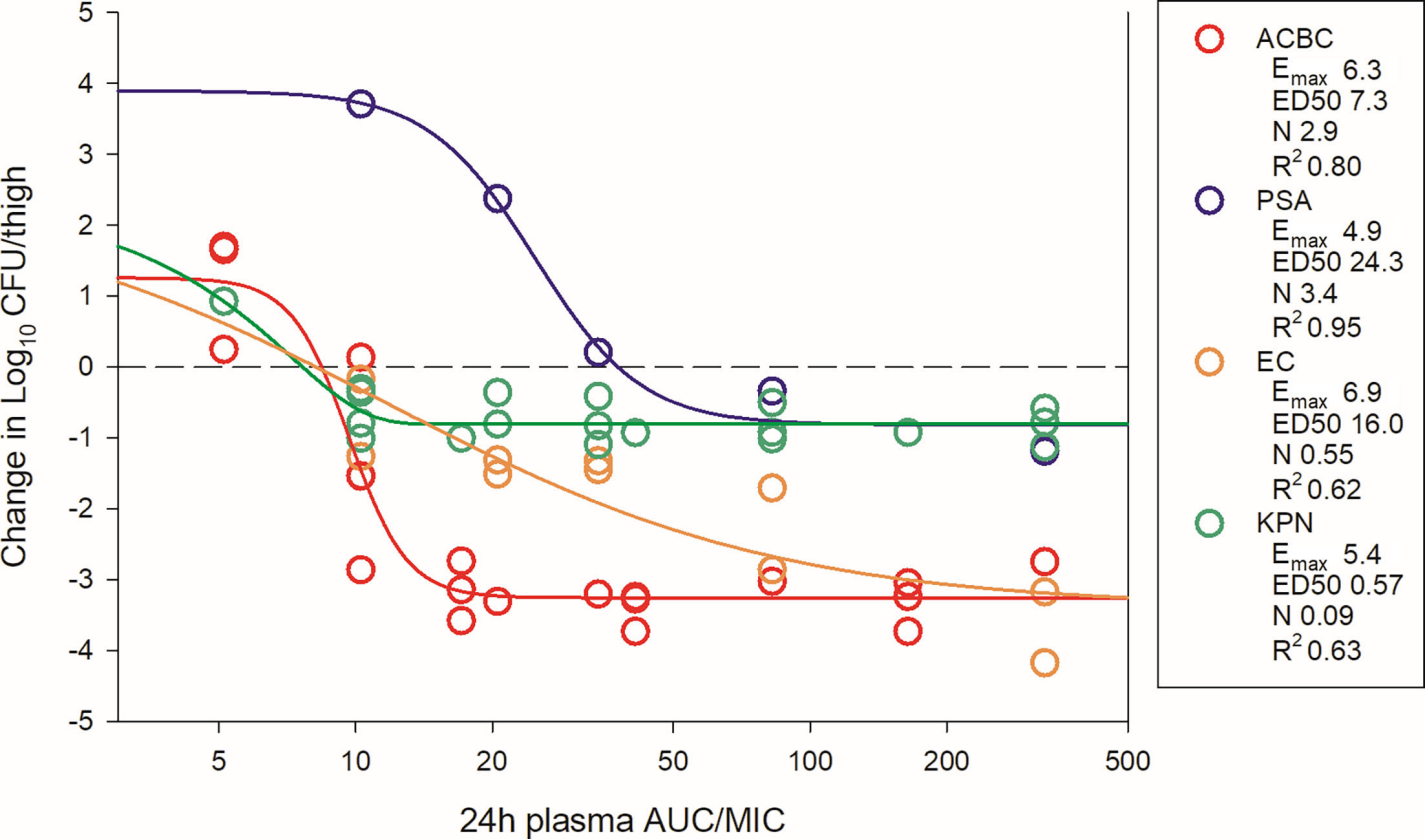
BRII-693 PK/PD exposure-response relationships against *E. coli*, *P. aeruginosa*, *K. pneumoniae*, and *A. baumannii* in the mouse thigh infection model. On the *x*-axis is the PK/PD index 24 h plasma total drug AUC/MIC, and on the *y*-axis is the therapeutic effect. PK/PD parameters *E*_max_ (maximal effect), ED_50_ (50% maximal effect), *N* (slope of the best-fit line), and *R*^2^ (coefficient of determination) are shown.

**Fig 5:**
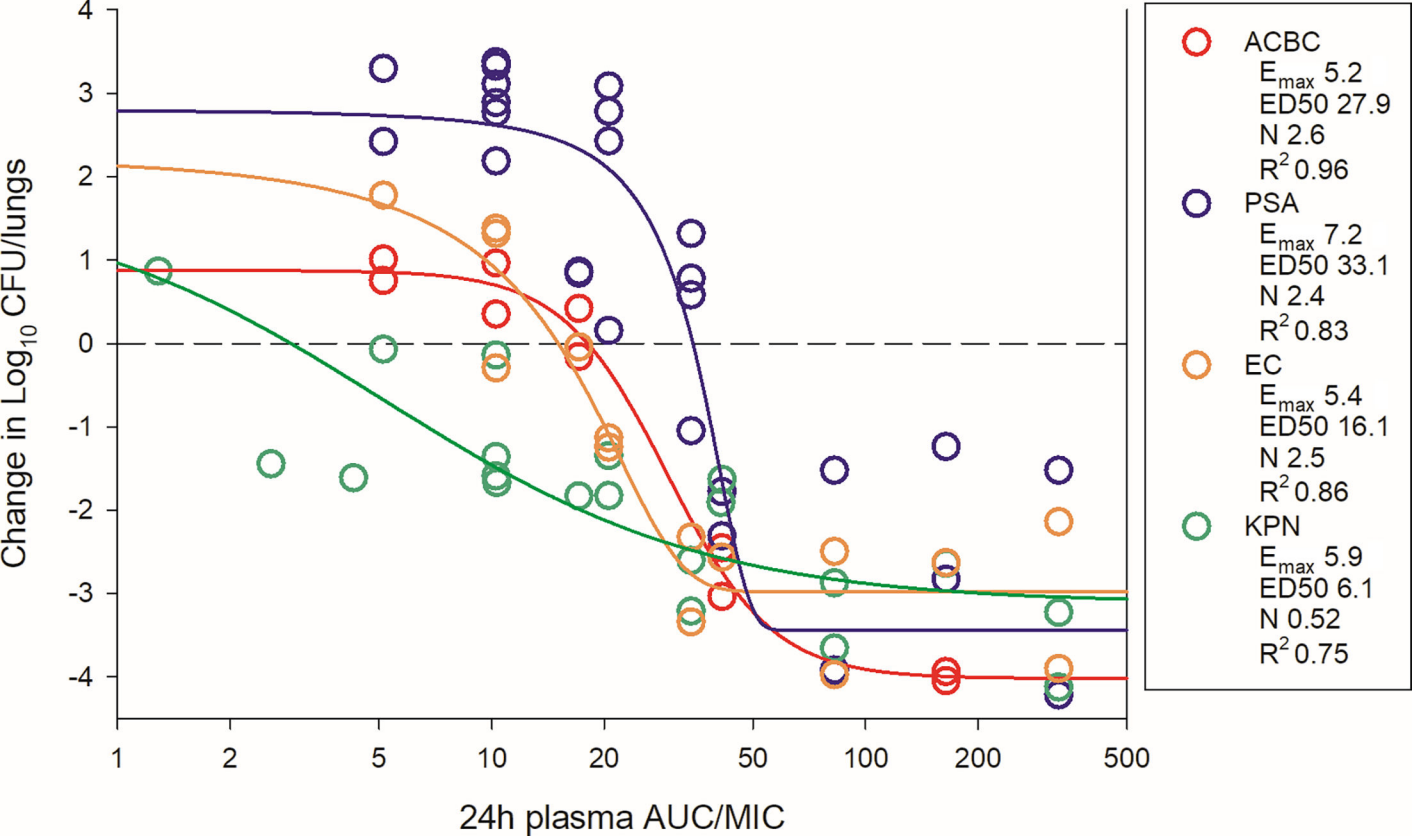
BRII-693 PK/PD exposure-response relationships against *E. coli*, *P. aeruginosa*, *K. pneumoniae*, and *A. baumannii* in the mouse lung infection model. On the *x*-axis is the PK/PD index 24 h plasma total drug AUC/MIC, and on the *y*-axis is the therapeutic effect. PK/PD parameters *E*_max_ (maximal effect), ED_50_ (50% maximal effect), *N* (slope of the best-fit line), and *R*^2^ (coefficient of determination) are shown.

**Fig 6:**
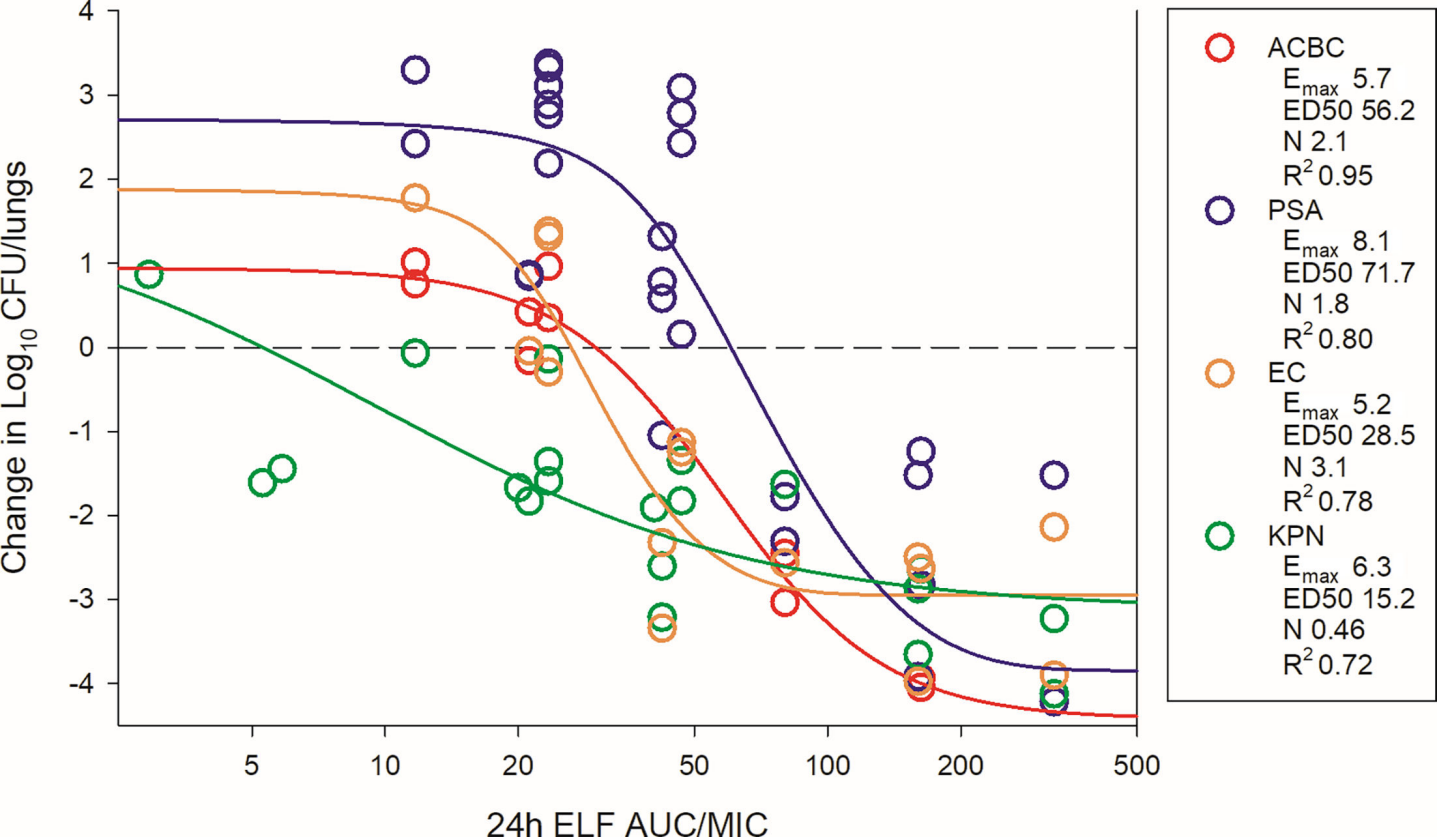
BRII-693 PK/PD exposure-response relationships against *E. coli*, *P. aeruginosa*, *K. pneumoniae*, and *A. baumannii* in the mouse lung infection model. On the *x*-axis is the PK/PD index 24 h ELF drug AUC/MIC, and on the *y*-axis is the therapeutic effect. PK/PD parameters *E*_max_ (maximal effect), ED_50_ (50% maximal effect), *N* (slope of the best-fit line), and *R*^2^ (coefficient of determination) are shown.

The dosing regimens were then converted into AUC_0–24_ estimates based on the animal pharmacokinetic data. The total drug AUC/MIC (tAUC/MIC) was calculated for each dose level-organism combination and plotted by pathogen group (Fig. 4 for thigh; Fig. 5 and 6 for lung). The 24 h tAUC/MIC was a robust predictor of efficacy with high *R*^2^ values. Exposure-response curves were steeper and lower (indicating greater cidal activity) in lung models compared to thigh models.

As shown in Table 2, the 24 h plasma tAUC/MIC values required to achieve stasis, 1-log kill, and 2-log kill were comparable among *A. baumannii*, *Escherichia coli*, and *K. pneumoniae*. For example, stasis was observed at 24 h plasma tAUC/MIC values between 1 and 19 in both thigh and lung infection models for these organisms. In contrast, *P. aeruginosa* required higher plasma tAUC/MIC for stasis, ranging from 20 to 44. Due to increased steepness and depth of the exposure-response relationship noted specifically in the lung model, 1- and 2-log kill targets occurred at nominal increases in 24 h plasma tAUC/MIC values. For example, 1-log kill in the lung model occurred at 24 h plasma tAUC/MIC <2 fold higher than stasis targets in 13 of 15 experiments, and 2-log kill in the lung model occurred at 24 h plasma tAUC/MIC <2 fold higher than stasis targets in 7 of 15 experiments. Across all organisms, maximal bacterial reduction in the lung model plateaued at 24 h plasma tAUC/MIC values of ~50.

ELF exposures are similarly represented in Table 2. Due to prolonged half-life and increased AUC within ELF, the ELF AUC/MIC targets were modestly higher than those observed in plasma.

## DISCUSSION

BRII-693 is a novel synthetic variant macrocyclic peptide antibiotic designed to treat multi-drug-resistant gram-negative bacterial infections, including those caused by *Enterobacterales* spp., *Pseudomonas aeruginosa*, and *Acinetobacter baumannii*. Importantly, BRII-693 is designed to mitigate the toxicities commonly associated with traditional polymyxin use. This study characterizes the PK/PD exposure-response relationships of BRII-693 using well-established immunocompromised mouse thigh and lung infection models. Prior dose-fractionation studies have established that both C_max_/MIC and AUC/MIC are predictive PK/PD indices for polymyxins, which exhibit concentration-dependent killing with limited post-antibiotic effects (15–22). Therefore, in the current studies, we examined and enumerated the exposure-response relationship for 24 h AUC/MIC and therapeutic effect, including traditional stasis and cidal endpoints. Indeed, AUC/MIC was a robust predictor of therapeutic efficacy across the different species and models (*R*^2^ 0.62–0.96). It should be noted that while protein binding was not included as part of the current studies, previous studies with BRII-693 have determined that the protein binding is approximately 36% in mice and 45% in humans (10). Therefore, the free drug AUC/MIC (fAUC/MIC) values are approximately 64% of the total AUC/MIC values reported in Table 2.

In the thigh infection model, cidal outcomes varied by species. *P. aeruginosa* and *K. pneumoniae* achieved maximal effects of 1-log kill, while *A. baumannii* and *E. coli* achieved a 3-log kill maximal effect. It is important to note that the *P. aeruginosa* data are based on a single strain. Additionally, we and others have consistently shown muted polymyxin activity against *K. pneumoniae* characterized by relatively flat exposure-response relationships and limited cidal activity (20, 21). The reason for this is not completely understood mechanistically. One proposed explanation is the presence of abundant extracellular capsule produced by many *K. pneumoniae* strains (20), which may impact drug efficacy. Further mechanistic studies into the exposure-response relationships for *K. pneumoniae* are likely warranted for this class.

In earlier studies, against select gram-negative strains, the polymyxin B 24 h plasma fAUC/MIC values associated with net stasis in the mouse thigh model have varied between 5 and 50 (16–18, 20, 21). For BRII-693, the PK/PD target in thigh studies was notably lower but still within this range with 24 h plasma fAUC/MIC values < 7 (adjusted for 36% protein binding), except for the single *P. aeruginosa* experiment. Cidal endpoints, such as 1-log kill, have not been consistently reported in prior animal model experiments.

In the lung infection model, BRII-693 demonstrated consistent and robust cidal activity across all tested organisms. The 1-log kill endpoint was associated with 24 h plasma tAUC/MIC values ranging from 20 to 50, which correspond to free drug fAUC/MIC values of 13–32. Maximal effect (3–4-log kill) was achieved at a 24 h plasma tAUC/MIC of 50 (free drug fAUC/MIC 32) for all organisms tested.

This level of consistency is notable, given that previous studies with polymyxin compounds have struggled to demonstrate reproducible efficacy based on PK/PD target exposures in mouse lung infection models (16, 20). From a drug development perspective, these findings of impressive and consistent cidal efficacy across heterogeneous groups of pathogens are encouraging, suggesting that BRII-693 may overcome some of the historical limitations of polymyxins in treating pulmonary infections. It is also striking that the current recommended dosing guidelines for colistin and polymyxin B suggest targeting a 24 h plasma total drug AUC/MIC of 50–100 to achieve 1-log bacterial kill (23). Interestingly, this same AUC/MIC range is strongly associated with maximal effect (3- to 4-log kill) observed for BRII-693 in murine lung infection models. These findings suggest that the PK/PD target exposure-response relationship for BRII-693 aligns with established benchmarks for polymyxin-class antibiotics. In the context of human PK data, MIC distribution, and safety studies for BRII-693, this supports the potential to design clinical dosing regimens that aim for maximal effect against key gram-negative pathogens by targeting a 24 h plasma AUC/MIC of 50, a value already used in clinical practice for this drug class.

Similar exposure-response relationships were observed when using ELF concentrations instead of plasma. However, the exposure-response curves for ELF were right-shifted, reflecting higher ELF drug exposures per mg/kg compared to plasma. Although C_max_ concentrations were higher in plasma, the AUC was higher within the ELF due to a longer elimination half-life within ELF. This prolonged residence time may partially explain the enhanced efficacy of BRII-693 in lung models compared to thigh models. If similar pharmacokinetics are confirmed in humans, this could represent a favorable characteristic for treating pneumonia, where sustained ELF exposure is critical.

Several limitations should be acknowledged. First, the study included a heterogeneous group of gram-negative pathogens from four species, but the number of strains per species was limited. Additionally, fewer studies were conducted in the thigh model compared to the lung model. This was intentional, as BRII-693 is being developed primarily for drug-resistant gram-negative pneumonia, and lung models were prioritized accordingly. Strain fitness in the lung model constrained inclusion. For example, although 10 strains of *A. baumannii* were tested, only two grew sufficiently in controls to be analyzed (data not shown). Given the encouraging results of this first comprehensive PK/PD study with BRII-693, further studies with increased strains and those with specific resistance determinants will be fruitful. Additionally, mouse pharmacokinetics were performed in uninfected animals, which can impact ELF kinetics. However, typically, drug concentrations are elevated in the infected state. Finally, toxicity was not formally assessed, although no overt signs of toxicity were observed in the mice.

In summary, BRII-693 demonstrated dose-dependent pharmacokinetics in both plasma and ELF across a wide dose range in mice. The PK/PD index of AUC/MIC was strongly correlated with therapeutic efficacy. Potent *in vivo* activity was noted for BRII-693 against diverse gram-negative pathogens (*E. coli*, *P. aeruginosa*, *K. pneumoniae*, and *A. baumannii*) in both thigh and lung infection models. In particular, the lung model consistently showed ≥2-log kill across all pathogen groups. Achieving a 24 h plasma tAUC/MIC of 50 (fAUC/MIC of 32) was associated with maximal effect of 3–4-log kill in the lung model, matching the exposure targets for approved polymyxins in clinical use. These results provide a translational foundation for designing human dosing regimens. Further development of BRII-693 is warranted, especially given the urgent need for novel polymyxin analogs that offer improved efficacy and reduced toxicity.

## MATERIALS AND METHODS

The materials and methods in this manuscript have been previously described in our publications (21, 24, 25) and reproduced here for the convenience of the reader.

### Organisms, media, and antibiotic

Three *E. coli*, six *P. aeruginosa*, four *K. pneumoniae*, and four *A. baumannii* were utilized (Table 1). Strains were chosen to vary the susceptibility to BRII-693 as much as possible while ensuring similar growth in untreated controls. All organisms were grown, subcultured, and quantified using Mueller-Hinton broth (MHB) and agar (Difco Laboratories, Detroit, MI). The BRII-693 test materials used for *in vitro* and *in vivo* studies were supplied by Brii Biosciences. Drug concentrations were prepared in 5% dextrose in water.

### *In vitro* susceptibility studies

The MICs for BRII-693 were determined using Clinical and Laboratory Standards Institute microdilution methods (26). All MIC assays were performed in duplicate on three separate occasions. The median MIC of replicate assays is reported and utilized in the PK/PD analysis.

### Murine thigh and lung models

Animals were maintained in accordance with the Association for Assessment and Accreditation of Laboratory Animal Care International (27). All animal studies were approved by the Animal Research Committees of the William S. Middleton Memorial VA Hospital and the University of Wisconsin. Six-week-old, specific pathogen-free, female ICR/Swiss mice weighing 24 to 27 g were used for all studies (Harlan Sprague-Dawley, Indianapolis, IN). Mice were rendered neutropenic (neutrophils <100/mm^3^) by injecting cyclophosphamide (Mead Johnson Pharmaceuticals, Evansville, IN) subcutaneously 4 days (150 mg/kg) and 1 day (100 mg/kg) before thigh infection. Broth cultures of freshly plated bacteria were grown to logarithmic phase overnight to an absorbance of 0.3 at 580 nm (Spectronic 88; Bausch and Lomb, Rochester, NY). After a 1:10 dilution into fresh MHB, bacterial counts of the inoculum ranged from 10^6.9^ to 10^7.1^ CFU/mL. Thigh infections with each of the isolates were produced by injection of 0.1 mL of inoculum into both thighs of isoflurane-anesthetized mice. Treatment with BRII-693 (200 µL per dose) was initiated 2 h after induction of infection and continued for 24 h, at which point the treatment groups and controls were sacrificed for CFU enumeration. Organism burden was quantified by CFU counts from serial dilutions of thigh homogenates.

The lung model infection was similar in that 6-week-old, specific pathogen-free, female ICR/Swiss mice weighing 24 to 27 g were used for all studies. Mice were rendered neutropenic by cyclophosphamide injection and logarithmic phase cultures used to infect the mice as described above. After a 1:10 dilution, bacterial counts of the inoculum ranged from 10^7.7^ to 10^7.9^ CFU/mL. Lung infection was produced by intranasal administration of 50 µL of the inoculum in isoflurane-anesthetized mice held upright to produce aspiration into the lungs. Treatment with BRII-693 was initiated 2 h after induction of infection and continued for 24 h, at which point the treatment groups and controls were sacrificed for CFU enumeration. Organism burden was quantified by CFU counts from serial dilutions of lung homogenates.

### BRII-693 plasma and ELF pharmacokinetics in mice

Single-dose plasma pharmacokinetics of BRII-693 were evaluated in mice. Dose levels of 6, 12, 25, and 50 mg/kg of BRII-693 were administered intraperitoneally (IP). Plasma was collected from groups of three mice per time point at 0.25, 0.5, 1, 2, 4, 6, and 8 h for drug concentration determination. Plasma was obtained from each animal by centrifugation of anticoagulated blood obtained by cardiac puncture. K2-EDTA was utilized as the anticoagulant. Plasma was stored at −70°C until analysis. Drug concentrations were determined using LC-MS/MS.

ELF pharmacokinetics were similarly obtained using the same doses and sampling time points. Bronchiolar alveolar lavage (BAL) was performed in groups of three mice per dose and time point, with a single administration of 1 mL of sterile saline immediately removed for assay. The recovered BAL fluid was then centrifuged to separate the alveolar macrophages (cell pellet) and the ELF (supernatant). BRII-693 and urea concentrations were determined concurrently in both BAL and plasma samples to allow for the calculation of ELF drug concentrations using urea correction methodology, as described by Rennard et al. (28). LC-MS/MS was used for urea measurement. Corrected ELF concentrations were calculated according to the following formula: [Drug]ELF = [Drug]BAL × ([Urea]plasma/[Urea]BAL).

Pharmacokinetic parameters, including the elimination half-life (T_1/2_), 24-h area under the drug concentration-time curve (AUC), and maximum drug concentration (C_max_), were calculated using non-compartmental methods. The half-life was determined by linear least-squares regression. The AUC was calculated from the mean concentrations using the trapezoidal rule. The C_max_ values were obtained directly from observed data. The pharmacokinetic estimates for dose levels that were not measured were calculated using linear interpolation for dose levels between those with measured kinetics and using linear extrapolation for dose levels above or below those with measured kinetics based upon the relative linearity of kinetics over the measured range (*R*^2^ = 0.95).

### PK/PD magnitude studies for BRII-693

Dose-response experiments using neutropenic thigh and lung infection models were performed as described in methods above. BRII-693 was administered to groups of four mice for thigh infection experiments and groups of three mice for lung infection experiments. Untreated and zero-hour controls were included in all experiments. The dose range consisted of 3, 6, 12, 25, and 50 mg/kg administered every 6 h by IP injection. After 24 h, the mice were euthanized, and CFU counts were determined in the thighs or lungs. The exposure-response relationships were examined using nonlinear least-squares multivariate regression (Hill equation), correlating the 24 h AUC/MIC with treatment efficacy as described by the following equation: *E* = (*E*_max_ X *D^N^*)/(ED_50_*^N^ – D^N^*), where *E* is the effector, in this case, the log change in CFU between treated mice and untreated controls after the 24 h period of study; *E*_max_ is the maximum effect; *D* is the 24 h PK/PD index magnitude (i.e., AUC/MIC); ED_50_ is the PK/PD exposure associated with 50% of *E*_max_; and *N* is the slope of the exposure-response curve (SigmaPlot version 13; Systat Software, San Jose, CA). The values for *E*_max_, ED_50_, and *N* were calculated using nonlinear least-squares regression. The coefficient of determination (*R*^2^) was used to assess the strength of the relationship between PK/PD index AUC/MIC and treatment effect. The 24 h AUC/MIC required for a static effect and cidal endpoints (1-log kill and 2-log kill) were determined by utilizing the plasma and ELF drug concentrations.
